# Addition of probenecid to beta-lactam antibiotics in the treatment of skin and soft tissue infections: a systematic review

**DOI:** 10.1093/jac/dkag376

**Published:** 2026-11-04

**Authors:** Claudette Hewitt, Martin John Llewelyn, Elizabeth Cross

**Affiliations:** Centre of Infection and Antimicrobial Research (CINAMR), Brighton and Sussex Medical, School, University of Brighton and University of Sussex, Brighton BN1 9PX, UK; Department of Infection, University Hospitals Sussex NHS Foundation Trust, Royal Sussex, County Hospital, Brighton BN2 5BE, UK; Centre of Infection and Antimicrobial Research (CINAMR), Brighton and Sussex Medical, School, University of Brighton and University of Sussex, Brighton BN1 9PX, UK; Department of Infection, University Hospitals Sussex NHS Foundation Trust, Royal Sussex, County Hospital, Brighton BN2 5BE, UK; Centre of Infection and Antimicrobial Research (CINAMR), Brighton and Sussex Medical, School, University of Brighton and University of Sussex, Brighton BN1 9PX, UK; Department of Infection, University Hospitals Sussex NHS Foundation Trust, Royal Sussex, County Hospital, Brighton BN2 5BE, UK

## Abstract

**Background and Objectives:**

Among patients presenting to hospital with skin and soft tissue infection (SSTI), up to 50% have already failed oral antibiotic treatment and 90% receive intravenous (IV) antibiotics.

Probenecid prolongs serum concentrations of beta-lactam antibiotics. Probenecid-boosted oral treatment may be an alternative to IV. Alternatively, probenecid could allow less frequent dosing of narrow-spectrum IV agents (e.g. cefazolin), reducing dependence on ceftriaxone in ambulatory settings. This systematic review evaluated the effectiveness and safety of probenecid combined with beta-lactam antibiotics for SSTI treatment.

**Methods:**

MEDLINE and EMBASE were searched through February 2026. Eligible studies assessed the addition of probenecid to beta-lactam therapy in human participants with SSTIs and reported clinical outcomes or adverse effects.

**Results:**

Nine studies (over 1600 participants) were included, most evaluating once-daily IV cefazolin plus probenecid versus ceftriaxone or more frequent cefazolin dosing. Treatment success, failure, relapse and hospital admission rates were similar between groups, indicating that probenecid-boosted cefazolin performs comparably to standard regimens while offering a narrower-spectrum alternative to ceftriaxone. Evidence for oral beta-lactams plus probenecid was more limited but did not suggest inferiority. Adverse events, inconsistently reported, were generally mild and predominantly gastrointestinal, with possibly higher rates in the probenecid-containing regimens.

**Conclusions:**

Current evidence suggests probenecid may enable once-daily IV cefazolin as an alternative to ceftriaxone for SSTI, supporting narrower-spectrum antibiotics in ambulatory care. Evidence for using oral beta-lactams plus probenecid remains limited. A robust RCT is needed to determine clinical non-inferiority and acceptability of adverse effects. If effective, this approach could reduce IV complications, healthcare attendance and costs.

## Introduction

Cellulitis is a bacterial infection affecting the skin and underlying soft tissue. In England, the condition is responsible for over 100 000 emergency hospital admissions each year, and incidence continues to rise, particularly among older adults.^1,2^ Up to 50% of patients presenting to the emergency department have already failed oral antibiotic treatment in the community, and 90% are then treated with intravenous (IV) antibiotics due to concerns that oral therapy may not achieve adequate tissue concentrations.^3,4^

This reliance on IV therapy means that large numbers of patients require hospital-based care, exposing them to the disruption, inconvenience and clinical risks associated with admission, while also placing a major financial burden on the healthcare system, with inpatient costs in the UK estimated at around £400 million annually.^5^

To reduce hospital admissions, IV antibiotics may instead be delivered through alternative community-based services, including ambulatory care clinics, emergency or urgent treatment centres, OPAT programmes and Hospital at Home schemes. Although these models are less expensive than inpatient admission, they have important limitations.

Firstly, they are still considerably costlier than oral antibiotic treatment. Secondly, access to these services is inequitable, varying by gender and socioeconomic status, with some groups, including people who inject drugs, facing additional barriers to access.^6^ Thirdly, IV treatment itself can lead to complications, including catheter-related infections and thrombophlebitis.^7^ Finally, to facilitate less frequent antibiotic dosing in the community, IV ceftriaxone is often used, which is a broad-spectrum antibiotic, listed by the WHO as a ‘watch’ agent because of its propensity to cause antimicrobial resistance.^8^ The widespread use of ceftriaxone in outpatient skin and soft tissue infection (SSTI) treatment hampers efforts to reduce use of ‘watch’ antibiotic agents.^9^

Probenecid has been used clinically since the 1940s. Given by mouth, it acts to reduce renal clearance of beta-lactam antibiotics and so can sustain concentrations and extend dosing intervals.^10–12^ With the introduction of long half-life drugs like ceftriaxone, its use diminished although it is still used in many countries for the treatment of hyperuricaemia and as an adjunct to antimicrobial therapy in selected infections such as syphilis and gonorrhoea.^13,14^

Combining probenecid with narrow-spectrum IV agents like cefazolin or even oral beta-lactam antibiotics could reduce the use of ceftriaxone for the treatment of SSTI. This review was carried out to evaluate the effectiveness and safety of probenecid combined with beta-lactam antibiotics for the treatment of SSTI.

## Patients and methods

### Search strategy and selection criteria

We followed the Preferred Reporting Items for Systematic Reviews and Meta-Analyses guidelines (Supplementary material, available as Supplementary data at *JAC* Online).^15^ We searched MEDLINE (via Ovid) and EMBASE using the search strategy outlined in Figure 1. A search of the Cochrane Library did not identify any additional eligible studies beyond those retrieved from MEDLINE and EMBASE. Studies evaluating the addition of probenecid to beta-lactam antibiotic therapy for the management of SSTI in human participants of any age were considered for inclusion. Only full-text, original research articles were included. No language restrictions or lower date limit was applied, and studies published up to February 2026 were eligible for inclusion. Studies were required to report treatment outcomes and/or adverse effects to be eligible for inclusion. The review protocol was prospectively registered on the PROSPERO database (registration number CRD420261290922).^16^

**Figure 1.**
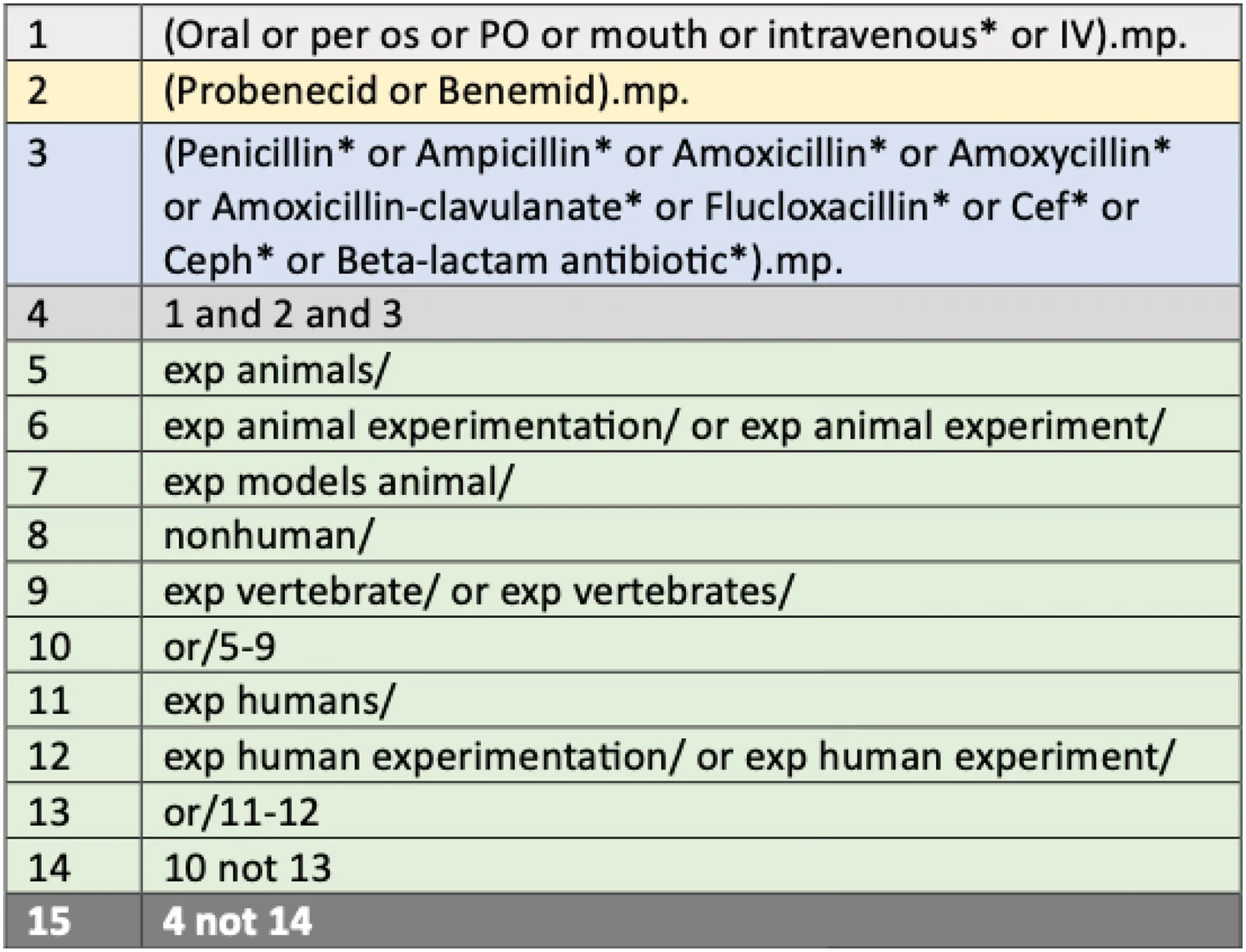
Search strategy used for the systematic review.

Search results were imported into Rayyan systematic review software, and duplicates were removed.^17^ Two authors (C.H. and E.C.) independently screened titles and abstracts against predefined inclusion and exclusion criteria. Full texts of potentially eligible studies were independently assessed by both reviewers. Disagreements were resolved through discussion and consensus. Backward citation searching of included studies and relevant review articles was undertaken to identify additional eligible studies.

### Data extraction

Data extraction was performed by one researcher (C.H.) using a standardized data extraction form, with independent cross-checking by a second reviewer (E.C.). Extracted data included publication characteristics (authors, journal and year of publication), study characteristics (study design, participant population, intervention, comparator and dosing schedules), clinical outcomes (including treatment success, failure and relapse) and reported adverse events or toxicity.

### Risk of bias

Risk of bias was assessed in accordance with Cochrane recommendations.^18,19^ Randomized controlled trials (RCTs) were assessed using the Cochrane Risk of Bias 2 (RoB 2) tool,^18^ while non-randomized studies (NRS) were assessed using the Risk Of Bias In Non-randomized Studies of Interventions (ROBINS-I) tool.^19^ Two reviewers (C.H. and E.C.) independently assessed risk of bias, with disagreements resolved through discussion and consensus.

### Data analysis

Given the anticipated heterogeneity in study design, interventions and outcome measures, meta-analysis was not considered appropriate. Instead, findings were synthesized narratively in accordance with the aims of the review. Treatment outcomes and adverse events were summarized descriptively, and relative risks of treatment failure were calculated where sufficient data were available.

## Results

### Study selection

Figure 2 outlines the study selection process. A total of 2102 references were identified through database searching, of which 45 duplicates were removed. Following title and abstract screening of 1840 records, 37 studies were assessed for full-text eligibility. Seven full texts were inaccessible. Six of these were published >60 years ago, lacked abstracts and appeared to be pharmacodynamic studies in healthy volunteers.^20–25^ The seventh, published in 1987 in a journal which no longer exists, could not be retrieved online or in print.^26^ Of the remaining 30 studies, 21 were excluded following full-text review. Seven were excluded because they involved the wrong population (healthy volunteer studies),^27–33^ six did not report relevant clinical outcomes^34–39^ and two did not investigate the intervention of interest independently.^40,41^ Additionally, five studies were excluded because only conference abstracts were available,^42–46^ and one article was a background article that did not present original research.^47^ Ultimately, nine studies met the inclusion criteria and were included in the review.

**Figure 2.**
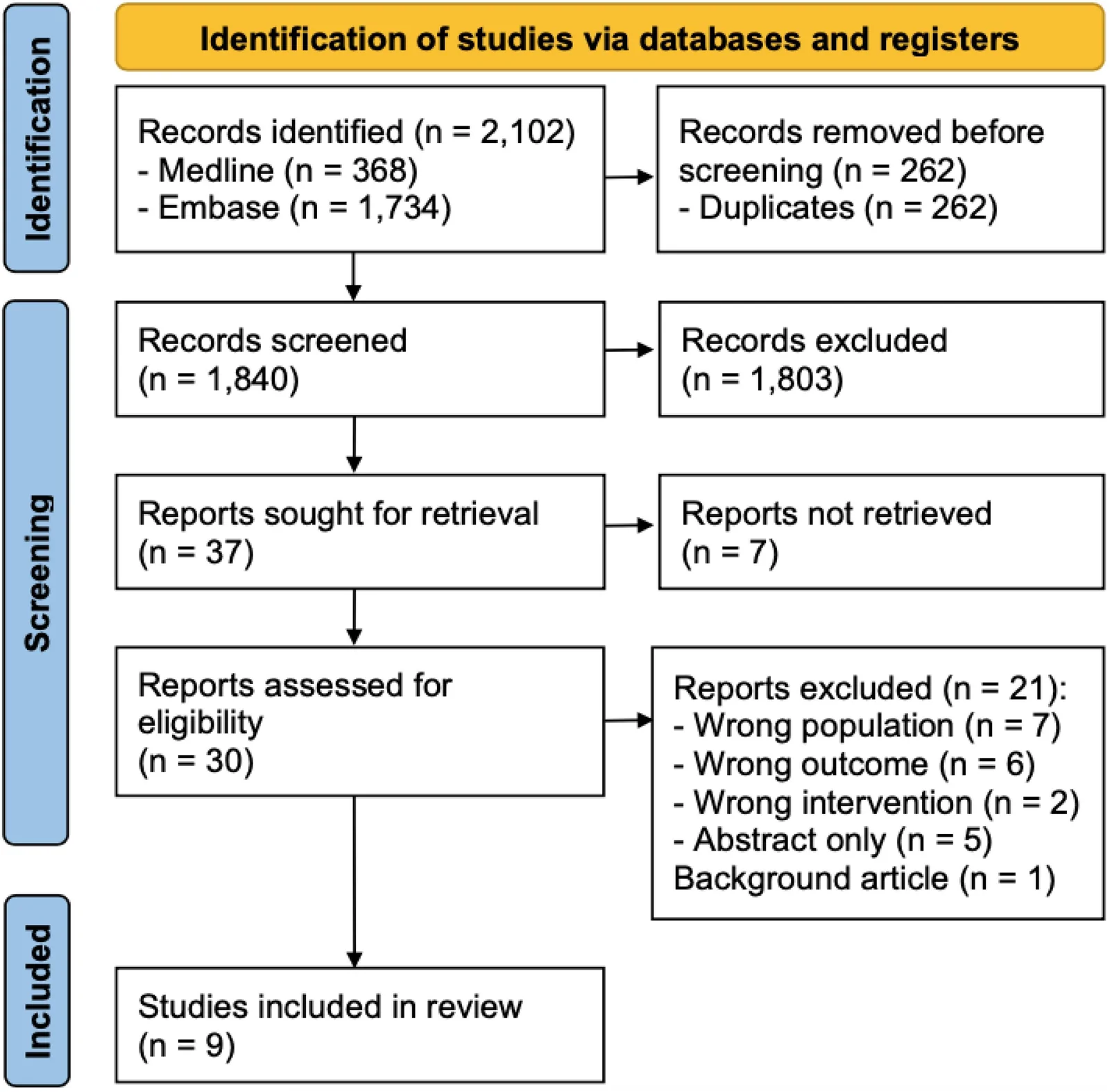
PRISMA 2020 flow diagram illustrating the identification, screening, eligibility assessment and inclusion of studies in the systematic review.

### Study characteristics

Nine studies involving more than 1600 participants were included, comprising three RCTs and six NRS published between 1996 and 2024. All nine studies had an arm consisting of once-daily IV cefazolin combined with probenecid. Comparator regimens included IV ceftriaxone (*n* = 3),^48–50^ more frequent cefazolin dosing (*n* = 3),^51–53^ continuous cefazolin infusion (*n* = 1)^54^ and oral antibiotic regimens (*n* = 2).^55,56^ In the two studies that included an oral antibiotic regimen, this was considered the intervention, as once-daily IV cefazolin plus probenecid was standard of care in these institutions. Only one study was conducted in a paediatric population. Study characteristics are summarized in Table 1.

**Table 1.** Study characteristics and efficacy results

Paper	Population	Design	Intervention (I)	Control (C)	Treatment failure (I vs C)	Admissions (I vs C)	Recurrence (I vs C)
**Cefazolin plus probenecid vs ceftriaxone**							
Brown *et al.* 1996	203 ED patients with cellulitis or soft tissue infection in Canada	Single-centre double-blind RCT	IV cefazolin 2 g q24h + PO probenecid 1 g q24h + oral antibiotics	IV ceftriaxone 2 g q24h + PO probenecid 1 g q24 h^a^ + oral antibiotics	8/98 (8.2%) vs 7/96 (7.3%) RR = 1.12 (95% CI 0.42–2.97)	3/98 (3.1%) vs 6/96 (6.3%) RR 0.49, 95% CI 0.14–1.71)	2/98 (2.0%) vs 0/96 (0.0%) RR (Haldane–anscombe): 4.90 (95% CI 0.24–100.7)
Chan *et al.* 2017	120 ED patients with cellulitis with lymphangitis or not improving with oral antibiotics in Singapore	Single-centre retrospective	IV cefazolin 2 g q24h + PO probenecid 1 g q24h	IV ceftriaxone 2g q24h	8/85 (9.4%) vs 4/35 (11.4%) RR: 0.82 (95% CI 0.26–2.56)^b^	8/85 (9.4%) vs 4/35 (11.4%) RR: 0.82 (95% CI 0.26–2.56)	No data
Grayson *et al.* 2002	134 patients with moderate-to-severe cellulitis requiring IV antibiotics in Australia	Multicentre double-blind RCT	IV cefazolin 2 g q24h + PO probenecid 1 g q24h	IV ceftriaxone 1g q24h	8/59 (13.6%) vs 2/57 (3.5%) RR: 3.87 (95% CI 0.86–17.43)	No data	2/48 (8.3%) vs 5/55 (9.1%) RR 0.46 (95% CI 0.10–2.12)
**Cefazolin plus probenecid vs more frequent cefazolin dosing**							
Garrett *et al.* 2012	332 patients with uncomplicated cellulitis requiring IV antibiotics in Australia	Multicentre, prospective observational	IV cefazolin 2 g q24h + PO probenecid 1 g q24h	IV cefazolin 2 g q12h	7/228 (3.1%) vs 10/104 (9.6%) RR: 0.32 (95% CI 0.13–0.82)	No data	No data
Khangura *et al.* 2007	269 paediatric ED patients with cellulitis in Canada	Single-centre retrospective	IV cefazolin q12h + PO probenecid (mean cefazolin dose 35.2 mg/kg +/− 11.0, probenecid dose not stated)	IV cefazolin q8h (mean dose 32.7 mg/kg +/− 13.2)	7/85 (8.2%) vs 12/39 (31%) RR: 0.27 (95% CI 0.11–0.63)	No data	No data
Landry *et al.* 2019	203 ED patients with uncomplicated cellulitis requiring IV antibiotics in Canada	Single centre retrospective	IV cefazolin 2 g q24h + PO probenecid 1 g q24h	IV cefazolin infusion (2 g loading + 6 g continuous over 24 h)	14/96 (14.6%) vs 8/106 (7.5%) RR: 1.93 (95% CI 0.85–4.40)^b^	No data	2/96 (2.1%) vs 4/106 (5.7%) RR 0.55 (95% CI 0.10–2.95)
Spina *et al.* 2003	26 adult patients suspected to have a bacterial infection requiring IV cefazolin for ≥5 days in Canada	Single-centre, prospective observational	IV cefazolin 1 g q24h + PO probenecid 500 mg q6h	IV cefazolin 2 g q8h	4/21 (19%) vs 1/5 (20%) RR: 0.95 (95% CI 0.13–6.76)^b^	No data	No data
**Cefazolin plus probenecid vs oral beta-lactams +/− probenecid**							
Bowhay *et al.* 2024	193 ED patients with moderately severe cellulitis in New Zealand	Single centre retrospective	Post-pathway change 69/70 (98.6%) had PO flucloxacillin 1 g q6h + PO probenecid 500 mg q6h^c^	Pre-pathway change 97/123 (78.9%) had IV cefazolin 2 g q24h + PO probenecid 1 g q24h	10/70 (14.3%) vs 19/123 (15.4%) RR: 0.93 (95% CI 0.46–1.88)	9/70 (12.9%) vs 15/123 (12.2%) RR = 1.05 (95% CI 0.59–1.98)	No data
Dalen *et al.* 2018	206 ED patients with mild-moderate SSTI in Canada	Multicentre double-blind RCT	PO Cefalexin 500 mg q6h^c^	IV cefazolin 2 g q24h + PO probenecid 1 g q24h	4/96 (4.2%) vs 6/99 (6.1%) RR: 0.69 (95% CI 0.20–2.36)	4/101 (4.0%) vs 1/102 (1.0%) RR: 4.04 (95% CI 0.46–35.57)	No data

CI, confidence interval; ED, emergency department; g, grams; h, hours; IV, intravenous; mg, milligrams; PO, oral; RCT, randomized control trial; RR, relative risk; SSTI, skin and soft tissue infection; vs, versus.

^a^The study authors reported that the pharmacokinetics of ceftriaxone were not significantly altered by concurrent administration of probenecid.

^b^Treatment success outcomes were converted to treatment failure.

^c^IV cefazolin plus probenecid was standard of care in these institutions; therefore, the oral regimen is the intervention.

### Risk of bias

Figure 3 summarizes the risk of bias across included studies. All six NRS were assessed as being at serious risk of bias, as they did not take appropriate measures to control for potential confounding and all but one of these studies was at moderate risk of bias for the measurement of outcomes.^49,51–55^ Among the three RCTs, one was at low risk of bias, one moderate and one high.^48,50,56^

**Figure 3.**
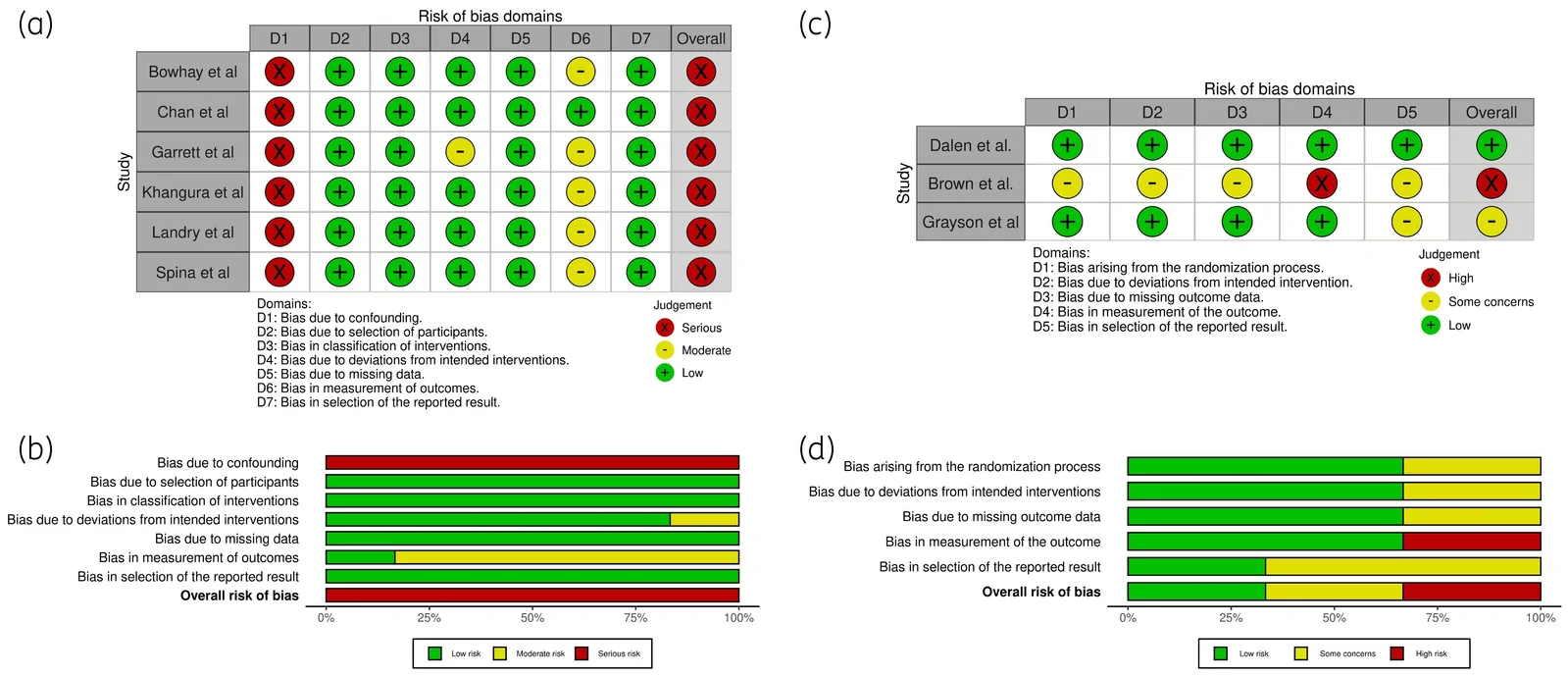
Summary of the risk of bias assessment for studies included within the review. (a) Risk of bias domains for individual non-randomized studies evaluated using the Risk of Bias in Non-randomised Interventional Studies (ROBIN-I) assessment tool. (b) Summary of risk of bias domains for non-randomized studies assessed using ROBIN-I. (c) Risk of bias domains for individual randomized studies evaluated using the Risk of Bias Assessment Tool version 2 (ROB-2). (d) Summary of risk of bias domains for randomized studies assessed using ROB-2.

### Clinical outcomes

Six studies reported on treatment success,^49–51,53,54,56^ seven on treatment failure,^48,50–52,54–56^ three on recurrence^48,50,54^ and four on readmissions.^48,49,55,56^ To facilitate comparison across studies, treatment success outcomes were converted to treatment failure outcomes (Table 1). This was possible because the outcome definitions allowed patients to be classified unambiguously as either treatment success or treatment failure, with no indeterminate categories, and minimal missing data.

Three studies (two RCTs and one NRS) compared once-daily IV cefazolin plus probenecid versus once-daily IV ceftriaxone.^48–50^ Treatment failure rates were between 3.5% and 13.6%, and there was no evidence of a difference between groups. Hospital admission rates were low in both groups with admission occurring in 3.1% versus 6.3% in the RCT by Brown *et al.* and 9.4% versus 11.4% in the NRS by Chan *et al.* (RR 0.49, 95% CI 0.14–1.71 and RR 0.82, 95% CI 0.26–2.56, respectively).^48,49^ Recurrence was uncommon, occurring in 2% versus 0% of participants in Brown *et al.* and 4.3% versus 9.1% in Grayson *et al.* (RR 4.90, 95% CI 0.24–100.7 by Haldane–Anscombe formula and RR 0.46, 95% CI 0.10–2.12, respectively).^48,50^

Four studies compared cefazolin plus probenecid versus more frequent cefazolin dosing.^51–54^ Garrett *et al.* and Khangura *et al.* reported lower treatment failure with probenecid-boosted regimens (RR 0.32, 95% CI 0.13–0.82 and RR 0.27, 95% CI 0.11–0.63, respectively).^51,52^ Garrett *et al*. used an algorithm-based approach to assign patients aged ≤75 without renal impairment to the probenecid-boosted regimen and those aged >75 or with renal impairment to the more frequent cefazolin regimen.^51^ Khangura *et al.* performed a retrospective notes review of antibiotic prescribing practices in a paediatric emergency department.^52^ Landry *et al.* and Spina *et al.* found no evidence of a difference in treatment failure between groups.^53,54^ None of the studies reported on readmission rates. Landry et al. reported on recurrence and found no significant difference between groups (2.1% versus 5.7%; RR 0.55, 95% CI 0.10–2.95).^54^

Only two studies reported on oral beta-lactam therapy either combined with probenecid or used alone.^55,56^ Bowhay *et al.* performed a retrospective cohort study that studied outcomes following a pathway change from IV cefazolin plus probenecid to predominantly oral flucloxacillin plus probenecid.^55^ There was no difference in treatment failure rates with pathway change (14.3% after pathway change versus 15.4% before; RR 0.93, 95% CI 0.46–1.88), and readmission rates were also similar (12.9% after pathway change versus 12.2% before, RR 1.05, 95% CI 0.59–1.98). Dalen *et al.* carried out an RCT comparing oral cephalexin to IV cefazolin plus probenecid in patients with mild to moderate SSTI in Canada. Treatment failure rates were similar in the oral group compared to the IV group (4.2% versus 6.1%; RR 1.45, 95% CI 0.42–4.99), and there was no significant difference in readmission rates (4% versus 1%; RR 4.04, 95% CI 0.46–35.57).^55^

Microbiological data were inconsistently reported across the included studies. Four trials (Brown *et al.*, Garrett *et al.*, Spina *et al.*, Bowhay *et al.*) collected no microbiological data,^43,48,53,55^ and two further studies (Landry *et al*., Dalen *et al.*) similarly performed no routine culture, although they excluded patients with a prior history of methicillin-resistant *Staphylococcus aureus* (MRSA) infection or colonization.^54,56^ Of the studies that did report culture data, Khangura *et al.* obtained wound cultures in only 16% of their paediatric cohort, with pathogens isolated in 12% of these samples, most commonly *S. aureus* and *Streptococcus pyogenes*, though results were not stratified by antimicrobial subgroup.^52^ Chan *et al.* reported two positive intra-operative samples taken from patients who developed abscesses requiring incision and drainage and yielding *Klebsiella pneumoniae* and MSSA, both susceptible to cefazolin and ceftriaxone.^49^ Grayson *et al.* provided the most extensive microbiological dataset, with pathogens isolated in 34 of 47 wound cultures (72%).^50^ Oxacillin-susceptible *S. aureus* was the most frequently recovered organism, either alone or in combination with streptococci or gram-negative species, and *Streptococcus* species were identified in seven cases. A single case of bacteraemia due to *Streptococcus mitis*-associated endocarditis resulted in study withdrawal. Among patients in this study who experienced treatment failure or recurrence, repeat cultures yielded pathogens in only a minority of cases.

### Side effects and toxicity

Adverse events were inconsistently reported but were generally mild and predominantly gastrointestinal in nature, as summarized in Table 2. Six studies reported adverse effects systematically in both treatment groups, with no evidence of significant differences between groups. Where numerical differences were observed, adverse events were more frequent in participants receiving cefazolin plus probenecid.^50,51,53^ Although Bowhay *et al.* reported antimicrobial changes due to intolerance in 3% and 6% of participants in the two probenecid-containing treatment arms,^55^ all other non-serious adverse events were managed without changing antimicrobial therapy. Three serious adverse events were reported across the studies and included one case of gastrointestinal intolerance requiring hospital admission in the cefazolin plus probenecid group,^50^ one case of antibiotic-associated interstitial nephritis in the ceftriaxone group^49^ and one case of a rash requiring hospital admission in the oral cephalexin without probenecid group.^56^

**Table 2.** Summary of adverse effects

Paper	Adverse effects (intervention vs control)	Description
**Cefazolin plus probenecid vs ceftriaxone**		
Brown *et al.* 1996	No data	
Chan *et al.* 2017	Not systematically recorded	*One patient from ceftriaxone arm admitted with antibiotic-related interstitial nephritis*
Grayson *et al.* 2002	14/67 (21%) vs 7/67 (10%), (RR: 2.00, 95% CI 0.86–4.67)	*Predominantly GI symptoms; 1 x serious in cefazolin + probenecid arm—admission to hospital due to severe vomiting + diarrhoea*
**Cefazolin plus probenecid vs more frequent cefazolin dosing**		
Garrett *et al.* 2012	33/213 (15%) vs 8/88 (9%), (RR: 1.71, 95% CI 0.82–3.55)	*Predominantly GI symptoms; no reactions necessitating discontinuation of study drugs*
Khangura *et al.* 2007	Not systematically recorded	*Only side effects stated were those of probenecid (5): nausea, vomiting, abdominal pain, difficulty swallowing and refusal of child to take probenecid*
Landry *et al.* 2019	4/96 (4%) vs 4/107 (4%) (RR: 1.12, 95% CI 0.29–4.32)	*Reactions were either rash or nausea*
Spina *et al.* 2003	9/21 (43%) vs 1/5 (20%) (RR: 2.14, 95% CI 0.35–13.22)	*Most common adverse event: fatigue (1 and 3, respectively)*
**Cefazolin plus probenecid vs oral beta-lactams +/− probenecid**		
Bowhay *et al.* 2024	4/70 (6%) vs 4/123 (3%) (RR 1.76, 95% CI 0.45–6.81)	*Recorded as ‘antimicrobial change due to intolerance’*
Dalen *et al.* 2018	5/101 (5%) vs 4/102 (4%), (RR 1.26, 95% CI 0.35–4.56)	*Predominantly GI symptoms; 1 x serious in cephalexin arm—rash requiring hospital admission. No other reactions necessitating discontinuation of study drugs*

## Discussion

This systematic review identified nine studies involving more than 1600 participants that compared IV cefazolin combined with probenecid to a range of other antibiotic treatment regimens, including IV cefazolin alone with alternate dosing, IV ceftriaxone and oral antibiotic regimens. Across studies, clinical outcomes, including treatment success, failure, recurrence and readmissions were mostly comparable between intervention and comparator groups.

Only two studies comparing cefazolin plus probenecid versus more frequent cefazolin dosing reported lower treatment failures with probenecid-boosted regimens.^51,52^ However, Garrett *et al*. used an algorithmic approach to assign older and more comorbid patients to the cefazolin-only group.^51^ Furthermore, the finding from Khangura *et al.* was based on retrospective analysis of a small subgroup and this was the only study to focus on a paediatric population.^52^

All three studies that compared once-daily IV cefazolin plus probenecid with ceftriaxone found no evidence of a difference in treatment success or failure.^48–50^ Cefazolin is a first-generation cephalosporin with a narrower spectrum of activity than ceftriaxone. However, its use in cellulitis outpatient/ambulatory care pathways has been limited by the need for more frequent dosing, whereas ceftriaxone is widely used because it can be administered once daily. The World Health Organisation’s Access, Watch, and Reserve (AWaRe) framework classifies ceftriaxone as a ‘Watch’ antibiotic that should be reserved for situations where narrower-spectrum ‘Access’ antibiotics such as cefazolin are unsuitable.^8^ Consequently, cefazolin plus probenecid may help to mitigate unnecessary broad-spectrum antibiotic exposure for patients with SSTI. Indeed, cefazolin plus probenecid is established as standard care within outpatient SSTI pathways in several countries, including Canada, Australia and New Zealand.^38,57^

Notably, the included studies compared cefazolin plus probenecid against once-daily ceftriaxone regimens. Where ceftriaxone is instead prescribed twice daily, as is common practice for some indications (e.g. MSSA cellulitis), cefazolin plus probenecid may offer potential advantages in reducing healthcare visits and associated costs. However, the greater opportunity to reduce healthcare utilization and cost may lie in the use of probenecid-boosted oral beta-lactam regimens as an alternative to IV therapy altogether.

Evidence supporting the use of probenecid alongside oral beta-lactam therapy in place of IV antibiotic treatment is more limited. Bowhay *et al.* reported no significant increase in treatment failure following a change in local practice from IV cefazolin plus probenecid to PO flucloxacillin plus probenecid.^55^ However, this retrospective before-and-after cohort study was at serious risk of bias because the analysis did not account for potential confounding variables, including disease severity, co-morbidities or changes in healthcare utilization during the COVID-19 pandemic. In addition, participants were allocated according to the time period in which they presented, rather than the treatment they actually received, resulting in substantial treatment crossover, with 21.1% of participants in the pre-implementation cohort receiving probenecid-boosted oral therapy. Despite these limitations, New Zealand has adopted oral flucloxacillin plus probenecid as part of national antibiotic prescribing guidelines.^57^

Dalen *et al.* conducted the double-blind, non-inferiority RCT that found oral beta-lactam therapy alone to be non-inferior to IV cefazolin plus probenecid.^56^ However, several limitations should be considered when interpreting these findings. First, the trial failed to achieve its planned sample size and was therefore underpowered, increasing the risk of incorrectly concluding non-inferiority. Second, patients who had received more than 24 h of antibiotic therapy prior to presentation were excluded, accounting for 40% of screened patients. This is important because observational studies suggest that up to 50% of patients presenting to ED with cellulitis have already failed oral antibiotic treatment in the community.^3,4^ Consequently, the findings may not be generalizable to the broader emergency department population. These results suggest that improved risk stratification tools may be required to identify patients who can be managed successfully with oral therapy alone and those who may benefit from adjunctive strategies such as probenecid-boosted antibiotic therapy.

The overall paucity of systematic microbiological sampling across these trials likely reflects the characteristically low diagnostic yield of wound and blood cultures in SSTI, a finding consistent with the wider literature on SSTI.^58^ Where cultures were performed, MSSA emerged as the dominant pathogen, with streptococcal species representing the next most frequent isolate. Resistant organisms such as MRSA and cefazolin-resistant Gram-negative pathogens were identified only sporadically. Notably, in the study by Grayson et al., clinical treatment failure or recurrence did not consistently correlate with positive repeat cultures, suggesting that microbiological confirmation may be of limited value in guiding management decisions once empirical therapy has been initiated.^50^

The findings of this review are consistent with the broader literature evaluating probenecid as a pharmacokinetic enhancer. Wilson *et al.* conducted a systematic review and meta-analysis of oral beta-lactam antibiotics combined with probenecid across multiple clinical indications.^59^ Although half of the included studies were healthy volunteer pharmacokinetic studies, the review demonstrated that probenecid consistently increased oral beta-lactam exposure. Furthermore, pooled analysis suggested improved clinical outcomes in gonococcal infections treated with probenecid-boosted oral beta-lactam therapy compared with beta-lactam therapy alone (relative risk of treatment failure 0.33, 95% CI 0.20–0.55; *I*^2^ 7%). While these findings cannot be directly extrapolated to SSTI, they provide biological plausibility for the use of probenecid as an adjunctive agent to boost oral beta-lactam levels and support further investigation in SSTI.

Adverse events were reported inconsistently across studies, limiting definitive conclusions regarding safety. However, the available evidence suggests that probenecid is generally well tolerated, with gastrointestinal symptoms representing the most commonly reported adverse effects. These findings are consistent with safety data from other patient populations receiving beta-lactam antibiotics in combination with probenecid.^59^ Future trials would need to determine whether the potential benefits of probenecid-boosted oral therapy, including reduced IV access complications, healthcare contact and broad-spectrum antibiotic exposure, outweigh any increase in adverse effects from a patient perspective. The practical and economic feasibility of this approach will also depend on local probenecid licensing and supply arrangements, which may introduce additional costs or governance burden in settings where it is not routinely available.

The potential for clinically significant drug–drug interactions should also be considered. Probenecid is known to alter the pharmacokinetics of several commonly prescribed medications.^60^ Careful consideration of these interactions will be important when assessing the feasibility of introducing probenecid-containing treatments into routine clinical practice.

Strengths of this review include the comprehensive and up-to-date evaluation of probenecid use in the treatment of SSTIs. Unlike the review by Wilson *et al*., which synthesized evidence across multiple infectious syndromes and included predominantly pharmacokinetic studies, our review focussed specifically on the clinical effectiveness and safety of probenecid-containing regimens for SSTIs, incorporating both IV and oral treatment strategies and recently published comparative studies. This provides the first disease-specific synthesis of the available clinical evidence for probenecid use in cellulitis and other SSTIs.

Limitations include the small number of eligible studies and the heterogeneity of the available evidence. Studies varied with respect to antibiotic regimens, treatment duration, study design and severity of SSTI, limiting direct comparison and precluding quantitative synthesis. The current evidence base also lacks consideration of the pharmacokinetic rationale for probenecid-boosted regimens, which may be clinically relevant given the time-dependent activity of beta-lactams.^53^ The optimal probenecid dose, frequency and timing relative to beta-lactam administration remain unclear, further underscoring the need for a well-designed RCT.

The evidence base also included NRS, several of which were at moderate to serious risk of bias, and many studies were underpowered to detect differences in uncommon outcomes such as recurrence and serious adverse events. Adverse event reporting was inconsistent across studies, further limiting comparisons of treatment safety.

A further important limitation is the absence of a standardized, validated definition of treatment failure across the included studies. Definitions ranged from a clinician-assessed change in antibiotic agent or route to readmission or need for surgical intervention (Table 1). As Yadav et al. have shown, treatment failure in SSTI is inconsistently defined in the literature, and a change in antibiotic therapy does not necessarily indicate that such a change was clinically required, since decisions may be influenced by clinician preference, patient expectation, or service factors rather than objective non-response.^61^ Furthermore, clinical improvement in cellulitis often follows a gradual trajectory, and perceived ‘failure’ may sometimes reflect a mismatch between expected and actual timing of response rather than true treatment inadequacy.^62^ This heterogeneity limits the comparability of treatment failure rates across studies and may overstate apparent differences, or equivalence, between regimens.

## Conclusion

Current evidence suggests probenecid may enable once-daily IV cefazolin as an alternative to ceftriaxone for SSTIs, supporting the use of narrower-spectrum antibiotics in ambulatory care settings. Evidence for oral beta-lactams plus probenecid replacing IV therapy remains limited. A well-designed RCT is required to assess non-inferiority of clinical outcomes and tolerability of adverse effects. If effective, this strategy could reduce IV complications, healthcare visits and overall costs.

## Supplementary Material

dkag376_Supplementary_Data

## Data Availability

The data underlying this article will be shared on reasonable request to the corresponding author.

## Supplementary data

Table 1 is available as Supplementary data at *JAC* Online.
