# Supplementary material for: Addition of probenecid to beta-lactam antibiotics in the treatment of skin and soft tissue infections: a systematic review
Source: J Antimicrob Chemother. 2026 Nov 4;81(12):dkag376. doi: 10.1093/jac/dkag376 (PMC13732567; doi:10.1093/jac/dkag376)
Supplement: Supplementary file 1 [file dkag376_supplementary_data.docx]

Supplementary Table 1: Definition of treatment failure used by each included paper

| Paper | Treatment failure definition |
| --- | --- |
| Brown et al 1996 | Lack of resolution of infection requiring hospital administration or change in antibiotic therapy |
| Chan et al 2017 | Hospital admission due to worsening cellulitis requiring further course of IV antibiotic, surgical incision and drainage of abscess or acute renal impairment |
| Grayson et al 2002 | Inadequate improvement that necessitated a change in antibiotic therapy |
| Garrett et al 2012 | Readmission to hospital as a result of cellulitis failing to improve after 3 or more days IV antibiotics or increasing skin breakdown or observations/ vital signs suggestive of clinical deterioration |
| Khangura et al 2007 | Failure of OPAT requiring hospital admission or abscess formation requiring incision and drainage |
| Landry et al 2019 | Cure defined as: Improvement of signs and symptoms as judged by ED physician permitting either discontinuation of antibiotics or oral stepdown |
| Spina et al 2003 | Treatment was successful if the patient was prescribed oral antibiotic therapy. Treatment failure occurred if the patient’s condition deteriorated |
| Bowhay et al 2024 | A change or escalation of therapy within 28 days of initial cellulitis diagnosis |
| Dalen et al 2018 | Hospital admission, change in antibiotic (not due to adverse event) or persistent/ worsening signs 72-96hrs after starting antibiotics in ED |

*: the pharmacokinetics of ceftriaxone are not significantly altered by concurrent administration of probenecid
